# Dissociative electron attachment to gold(I)-based compounds: 4,5-dichloro-1,3-diethyl-imidazolylidene trifluoromethyl gold(I)

**DOI:** 10.3389/fchem.2023.1028008

**Published:** 2023-06-19

**Authors:** Maria Pintea, Nigel Mason, Anna Peiró-Franch, Ewan Clark, Kushal Samanta, Cristiano Glessi, Inga Lena Schmidtke, Thomas Luxford

**Affiliations:** ^1^ School of Physical Sciences, University of Kent, Canterbury, United Kingdom; ^2^ Department of Chemistry, University of Oslo, Oslo, Norway; ^3^ Department of Chemistry, J. Heyrovský Institute of Physical Chemistry of the Czech Academy of Sciences, Prague, Czechia

**Keywords:** dissociative electron attachment, gold imidazolyl compounds, focused electron beam deposition, XRD, gold precursors

## Abstract

With the use of proton-NMR and powder XRD (XRPD) studies, the suitability of specific Au-focused electron beam induced deposition (FEBID) precursors has been investigated with low electron energy, structure, excited states and resonances, structural crystal modifications, flexibility, and vaporization level. 4,5-Dichloro-1,3-diethyl-imidazolylidene trifluoromethyl gold(I) is a compound that is a uniquely designed precursor to meet the needs of focused electron beam-induced deposition at the nanostructure level, which proves its capability in creating high purity structures, and its growing importance in other AuIm_x_ and AuCl_n_B (where x and n are the number of radicals, B = CH, CH_3_, or Br) compounds in the radiation cancer therapy increases the efforts to design more suitable bonds in processes of SEM (scanning electron microscopy) deposition and in gas-phase studies. The investigation performed of its powder shape using the XRPD XPERT^3^ panalytical diffractometer based on CoK_α_ lines shows changes to its structure with change in temperature, level of vacuum, and light; the sensitivity of this compound makes it highly interesting in particular to the radiation research. Used in FEBID, though its smaller number of C, H, and O atoms has lower levels of C contamination in the structures and on the surface, it replaces these bonds with C–Cl and C–N bonds that have lower bond-breaking energy. However, it still needs an extra purification step in the deposition process, either H_2_O, O_2_, or H jets.

## Introduction

Increased importance of gold(I) compounds in the pharmaceutical drug development and cancer studies is observed, and thus the gold(I) compounds are developed from the synthesis step for quantum simulations and molecular dynamics analysis, as in our case, or for catalyzed reactions and reductive elimination/migratory insertion reactions (Gil-Rubio and Vicente, 2015). The most worth mentioning application of the gold(I) compounds is the inhibition of bacteria, such as *Escherichia coli* (Galassi et al., 2015), through bonding of the DHFR (dihydrofolate reductase) to C, F, or P of the gold compound, where a high reduction in the level of DHFR in solution with gold(I) compared to non-gold(I) is observed. A level of reduction of 0.1 from 2.2 to 2.1 of denaturated DHFR and 1.2 to 1 of the native DHFR is observed with inhibitory constants of 2.25 μM, 1.1 μM, and 8.63 μM for 4-benzoic acid-diphenyl-phosphene gold(I) chloride, 2-benzoic acid-diphenyl-phosphane gold(I) chloride, and 4,5-dichloroimidazolato-N-triphenylphosphine–gold(I), respectively, extending the use of the gold(I) compounds to the treatment of inflammatory infections, pneumonia, *E. coli*, and cancer. In the cancer research, the developments of the gold (III) and gold(I) compounds have opened new ways of treating, inhibiting, and preventing cancer development through the design of new precursors. The clinical trials (Malet-Martino and Martino, 2002) from 1994 of the aurofin compound (2,3,4,6-tetra-O-acetyl-b-1-D-thipyranosato-S-(triphenylphosphine)gold(I)) initially used in the treatment of rheumatoid arthritis (Mirzadeh et al., 2019) revealed new ways of inhibiting cysteine (Cys), seleno-cysteine (Sec), glutathione reductase (GR), mitochondrial thioredoxin reductase (TrxR2), and cytosolic thioredoxin reductase (TrxR1) and opened new paths in the design of new monodentate phosphine precursors. Heterometallic fac-[Re (bipy) (CO_3_) (L-AuPPh_3_)]^+^, where L = imidazole, alkynyl-imidazole, and alkynyl-pyridine in human lung cancer cell treatments (Fernández-Moreira et al., 2014) show values of IC_50_ of ∼50 times lower than the Re(I) only complexes, obtaining a shift in the localization from the mitochondria to the nucleus with the increase in the concentration of the compound to μM, removing the cytoplasmatic staining with the accumulation in the mitochondria. Other studies involving the usage of coinage metal complexes (Schuh et al., 2012; Tan et al., 2010; Nobili et al., 2010) study the use of Au(I) NHC complexes against ovarian cancer cells (A2780S) and cisplatin resistant cells (A278R) compared to the non-tumoral kidney HEK-293T cells with obtained inhibition of cell growth IC_50_ values from 2 to 30 μM. Compared to the aurofin precursor, the Au(I) NHC compounds do not lead to the oxidation of the HEK-293T cells targeting only the TrxR1 and TrxR2 enzymes. A high number of Au precursors have been studied as inhibitors in cancer treatment (Mármol et al., 2019; González-Rubio et al., 2022; Zou et al., 2015) over the past few years. Compounds such as Au (xant)PEt_3_ and Au (dedc)PEt_3_ (Mármol et al., 2019) present higher inhibition rates compared to the aurofin and AuCl(PR_3_) exhibits values of IC_50_ between 4.2 and 5.2 μM in targeting colon carcinoma cells. The bis-chelated gold(I) bisphosphane (2,3-bis(tert-butyl (methyl) phosphino) quinoxaline) (Mármol et al., 2019) has been found to be a promising drug for cancer therapy to inhibit cysteine (Cys), seleno-cysteine (Sec), glutathione reductase (GR), and TrxR. Au compounds containing H_2_TPP (5,10,15,20-tetraphenyl porphyrin) and dppe (1,2-bis (diphenyl phosphane) ethane), Au (TPP)Cl and Au (dppe)_2_Cl (Zou et al., 2015) are targeting cancer through reduction of Au(III)—Au(I).

Through the use of the velocity map imaging technique and dissociative electron attachment (DEA) mass spectroscopy studies, employed in multiple analyses involving Au compounds or compounds of gold substrates, we determine the fragmentation pathways with implications to focused electron beam deposition. At 157 nm, the velocity map imaging study of diatomic gold in combination with the density functional theory (DFT) and *ab initio* calculations brings insight into the dynamics of the Au–Au vibrational and excitational modes, bonding between species with d-electrons valence and the branching ratios for Au 5d^9^6s^2^ (^2^D_3/2_) and Au 5d^9^6s^2^ (^2^D_5/2_) (Gil-Rubio and Vicente, 2015). The optical absorption spectra of Au in vapor form show the allowed transition states between 211 and 229 nm from ^1^Π_u_ (II) to X^1^Σ_g_^+^ and isolates two dissociation processes, first one at a photon energy of 2.301–2.311 eV for Au 5d^10^6s^2^ (^2^D_5/2_) and the second one at 3.437–3.447 eV for Au 5d^10^6s^2^(^2^S_1/2_) + Au 5d^9^6s^2^ (^2^D_5/2_), showing particularity for gold cluster processes and the presence of the 6 s orbitals combined with the relativistic effects of the s electrons.

In nanotechnology applications, the assisted deposition of Au compounds has been performed successfully by Shawrav et al. (2016) with H_2_O as oxidative enhancer resulting pure Au nanostructures with a resistivity of 8.8 μΩcm with 91% purity of the structure. The Au content of the nanostructures resulting from the focused electron beam-induced deposition of Me_2_Au (tfac) was improved to reach values of 72% through the refining of the electron beam parameters and further to hit high purity levels of ∼90% through the plasma-assisted structure post-processing (Belić et al., 2017). Chien et al. (2021) report a carbon content of up to 60% in their Au-deposited nanostructures through their newly developed localized surface plasmon resonance measurement (built to enhance structure content reading) and a reduction of 20% of the carbon content through the H_2_O treatment of the nanostructures. In the normal non-assisted deposition of CF_3_-Au containing compounds (Carden et al., 2018; Carden et al., 2019), values of the Au content in the deposits of 22% in the case of CF_3_AuCNMe and 14% for CF_3_AuCNBu (Carden et al., 2019) were obtained with values of decomposition and sublimation temperatures of the two compounds evaluated at 51°C and 80°C (CF_3_AuCNMe) and 39°C and 126°C (CF_3_AuCNBu). CF_3_–Au containing precursors are known to have very good sublimation and decomposition temperatures becoming highly sought precursors for FEBID deposition (Hagen et al., 2008; Utke et al., 2008; Botman et al., 2009; Thorman et al., 2015), though the lower levels of the Au content and high C contamination (>60 at%) are indications of the need of a post-processing treatment or assisted deposition. Me_2_Au(Acac) presents comparable results when deposited and annealed at 100°C–300°C, forming structures close to 14 nm in size (Puydinger dos Santos et al., 2018), but at the same time reducing the carbon content at 300°C under H_2_ jet to almost 0% and removing it out of the lattice through heating. Kuhness et al. (2021) report the growth of AuC_x_ nanopillar results of FEBID of Me_2_Au (acac) with a height of 2 µm for the development of 3D plasmonic gold nanoantennae, as one of the many applications of induced chemistry at the nanoscale. The focus is indeed on the composition of the nanopillars that are further annealed (300°C) and purified using H_2_O jets at room temperature. The growth of nanoantennae and nanopillars have based a new lithographic method on the focused electron beam-induced process (FEBIP) by cooling the substrate and thin films to lower than 0°C and further irradiated using e-beams to form structures (Zhao et al., 2019)*,* or more sophisticated methods, such as GIS (gas injection systems) and computer-assisted deposition for the creation of highly complex and accurate 3D nanostructures (Fowlkes et al., 2018). The same methods have been applied to growing carbon nanotubes (Brintlinger et al., 2005), carbene nanostructures (Furst and Cazin, 2010; Johnson, 2016; Glessi et al., 2021), and cold ice organic nanostructures (Zhao et al., 2019). Nanostructures have been printed by Magén et al. (2021) using Au-based compounds in reactive atmospheres (Winkler et al., 2017) with very successful outcomes.

## Experimental section

### VsMI/mass spectroscopy

**FIGURE 1:**
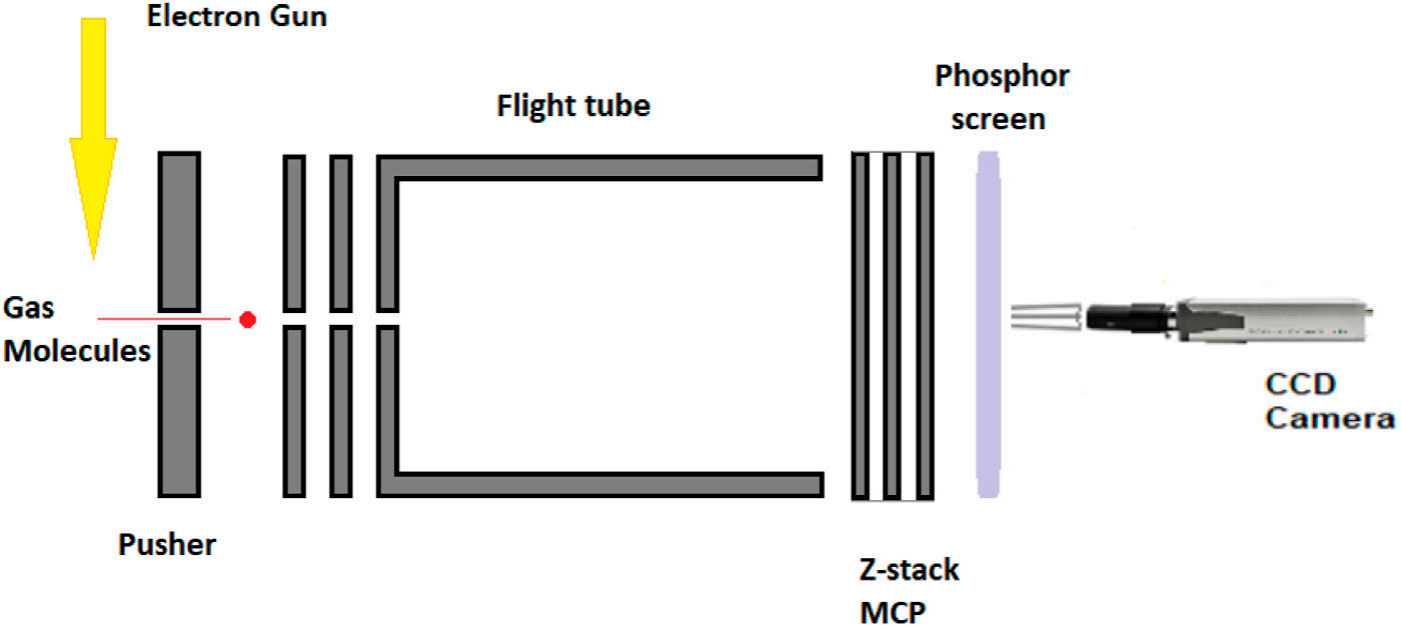
Velocity sliced map imaging (VsMI) detector assembly.

The experimental equipment consists of a high-vacuum chamber with pressures in the range of 10^−6^mbar helped by an Alcatel vacuum pump backed by a Pfeiffer Duo 6 backing pump. An electron gun is mounted on the top flange of the chamber intersecting at 90° of the molecular beam and in-line with the electron gun, and a three-plate Chevron pattern microchannel plate (MCP) detector. Puller, pusher, and flight tube assemblies (Figure 1) are connected with the detector for guiding the negatively charged ions to the phosphor screen. A charged-coupled device (CCD) industrial camera is used for capturing the ions accelerated at different velocities to the phosphor screen. A pair of Helmholtz coils is placed on the top and bottom of the chamber with the purpose of creating a magnetic field with values up to 80 Gauss that controls the guide path of the particles (ions/molecular fragments and electrons). The simple assembly, electron gun, the detector assembly (MCP), the flight tube, the phosphor screen, and the CCD camera for imaging the negative fragments, is helped by a 200-ns extractor/slicer that would physically select the inner slice of the Newton sphere of ions of a specific time length that are imaged by using the camera and detected by using MCP data acquisition modules for velocity discrimination. Similar set-ups (Prabhudesai et al., 2014; Bull et al., 2014; Gope et al., 2016) have been used at Tata Institute, India, and J. Heyrovský Institute of Physical Chemistry, Czech Republic (Nag et al., 2019), for imaging negative ions.

The increase in the number of detector’s plates reduces the aberration of the equipment and improves the energy range. The velocity sliced map imaging (VsMI) in Figure 1 assembly uses an energy range of 0–50 eV, specifically for low electron energy applications. The phosphor screen is employing a thin tungsten with a >98% transmission rate foil mounted on a brass ring. The detection of the negative ions is calibrated against a system of two molecules, namely, O_2_ at 6.5 eV and CO_2_, while the electron energy scale is zeroed with the signal and current calibration. The kinetic energy spectrum and angular distribution follow the same rules and should be less than 0.1% of the O_2_ and CO_2_ spectra. More detailed presentation of equipment and functioning has been given by Nag et al. (2019).

### XRD (X-ray powder diffraction)

To determine the structural characteristics of the crystalline sample XRD, measurements were taken using a XPERT^3^ panalytical diffractometer based on CoK_α_ with a time step of 150 s/step and a step size of 0.0167. The angle of diffraction is 5–80 ⁰ at a rate of 40 kV and 40 mA. The measurements were acquired over the duration of 1.5 h. The diffraction-specific wavelength is set to a value of 1.5406 Å.

### Single-crystal XRD

**FIGURE 2:**
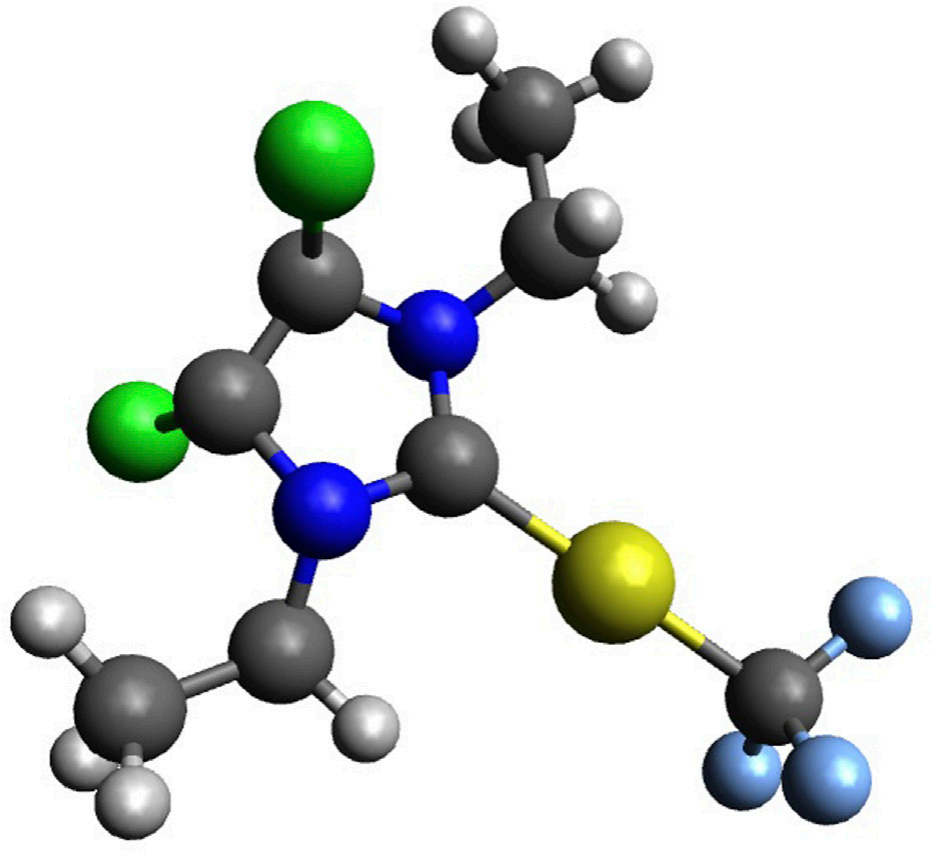
4,5-Dichloro-1,3-diethyl-imidazolylidene trifluoromethyl gold(I) (Au, yellow; N, blue; H, white; C, grey; F, light blue; and Cl, green).

10 mg of the 4,5-dichloro 1,3-diethyl imidazolylidene trifluoromethyl gold(I) complex (Figure 2) was dissolved in dichloromethane (20 mg/mL), followed by slow addition of pentane/hexane (dichloromethane: pentane/hexane is 5:1 vol/vol) onto the dichloromethane layer. The layered solutions were kept in the dark for crystallization for over 2 weeks at room temperature. Plate-shaped crystals of the trifluoromethyl gold(I) complex were obtained, hand-picked, and subjected to structure determinations by X-ray diffraction analysis.

### NMR (nuclear magnetic resonance)

The proton NMR (^1^H NMR) data acquisition was carried out using a JEOL ECS 400 MHz NMR spectrometer at 25°C with a sensitivity of 280 (0.1% ethyl benzene) for ^1^H and ^19^F, with an automatic Bruker SampleXpress sample charger run by using a 500 MHz electric DC motor having a 60 sample carousel controlled using ICON-NMR software and equipped with barcode reader registration, with the samples being kept at a temperature between 5 and 30°C and a separate cryo-fit mounting kit for sample cooling. The sample charger and sample unit were both controlled by the Bruker Avance III 400 MHz controller unit.

The sample (∼5 mg) was dissolved in 1-cm^3^ wet CDCl_3_ under atmospheric conditions; no special precautions were taken other than that, and the sample was initially transferred into the NMR tube in an Ar glove box.

### Synthesis of the gold(I) compound

**FIGURE 3:**
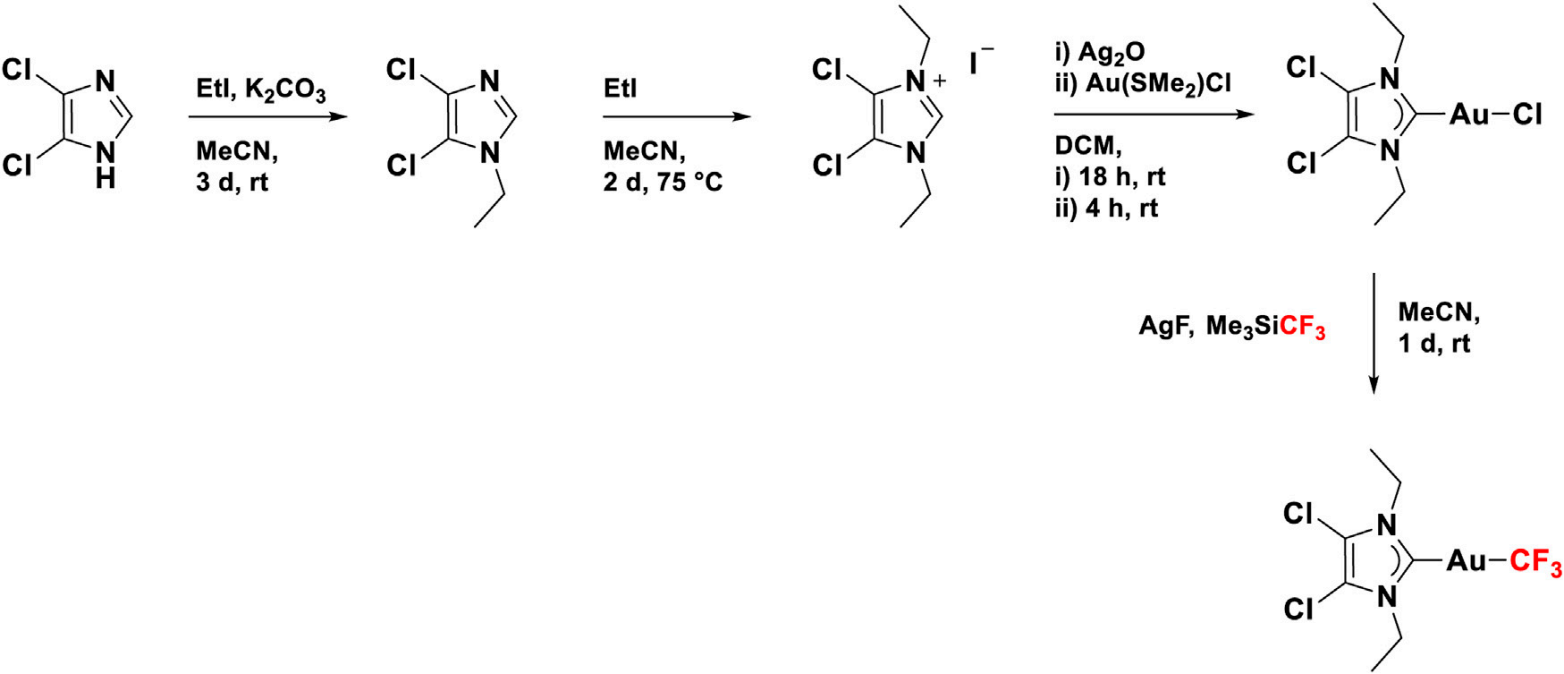
Synthesis route for 4,5-dichloro-1,3-diethyl-imidazolylidene trifluoromethyl gold(I).

Gold(I) NHC complexes are a class of compounds that are widely known and studied in chemistry for their versatility, among others in catalysis (Marion and Nolan, 2008), biomedicine (Porchia et al., 2018), and photochemistry (Longevial et al., 2016). The most important characteristic of the NHC ligand is the presence of carbene carbon, which is stabilized by two neighboring nitrogen atoms (Hopkinson et al., 2014). Due to their popularity, several ways for the synthesis of gold(I) NHC complexes have been reported (Wang and Lin, 1998), which makes these systems easily accessible and adaptable to required needs. The gold(I) NHC complex (Glessi et al., 2021) investigated in this work was synthesized following a reported literature procedure, which is illustrated in Figure 3. Starting from 4,5-chloroimidazole, the desired NHC ligand precursor was obtained as a salt in a yield of 94% through two sequential alkylation reactions using ethyl iodide (Solovyev et al., 2010). By reacting the NHC ligand precursor with silver oxide, the respective silver complex was formed *in situ*, which underwent a trans-metalation reaction upon the addition of one equivalent of the gold precursor Au(SMe_2_)Cl (Levchenko et al., 2020). The resulting gold NHC chloride complex was isolated in a yield of 92%. In the last step, the title compound was synthesized through another silver-mediated trans-metalation reaction. The active silver species AgCF_3_ is formed *in situ* from the reaction of silver fluoride with Me_3_SiCF_3_ and exchanges the chloro ligand with a CF_3_ group when Au(NHC)Cl is added, yielding the desired Au(NHC)CF_3_ complex as a colorless solid (66%) (Blaya et al., 2014a).

## Computational details

The simulations of the structure of C_8_H_10_Cl_2_N_2_AuCF_3_ have been run at the DFT/B3LYP level, making use of the full orbital populations and natural bond orbitals using a B3LYP/Def2-TZVPP basis set. The excited state calculations have been run using TDDFT. The crystal structure and slab for XRD simulations were built using Vesta and Avogadro software, and the single crystal structure modeling was carried out using OLEX2 crystallographic software based on the experimental data input.

## Results and discussion

### Structure characterization

**FIGURE 4:**
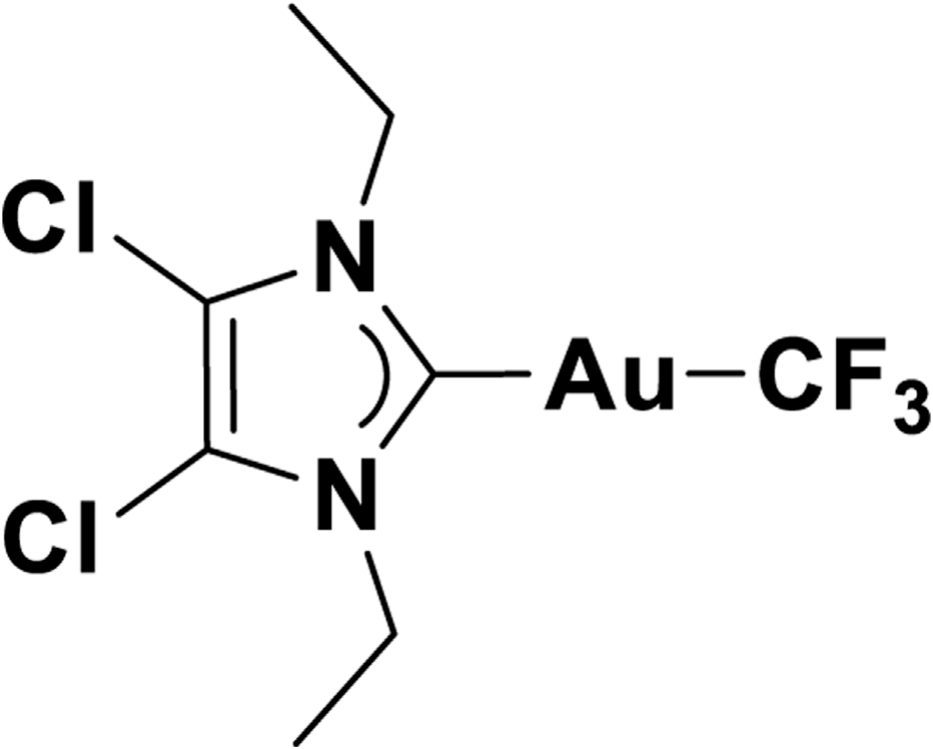
Schematics of 4,5-dichloro-1,3-diethyl-imidazolylidene trifluoromethyl gold(I).

4,5-Dichloro-1,3-diethyl-imidazolylidene trifluoromethyl gold(I) is a gold compound synthesized by the Chemistry Department of the University of Oslo. The empirical formula of the compound is C_8_H_10_Cl_2_N_2_AuF_3_, and it has a mass of 459.05. The schematic of the compound is illustrated in Figure 4.

A very important characteristic of our molecular dynamic simulations and cross-checking of our experimental results is the bond’s distance to C, Cl, N, and H and bond angles to C, Cl, N, and H. Multiple sources of trifluoromethyl gold(I) (Hasan et al., 1999; Chang et al., 2015; Ye et al., 2018) present the bond distances of the gold(I) compounds as 0.05 Å higher than the gold (II) compounds. In the study by Gil-Rubio and Vicente (2015), the bond distances for the most common gold(I) compounds are experimentally determined with values in the range of ∼2.04 Å as presented in Table 1.

**TABLE 1:** L–Au–CF_3_ bond distances (Gil-Rubio and Vicente, 2015).

Compound	d (Au–C)/Å, X = F
Ph_3_P–Au–CX_3_	2.045
IPr–Au–CX_3_	2.042; 2.030

**FIGURE 5:**
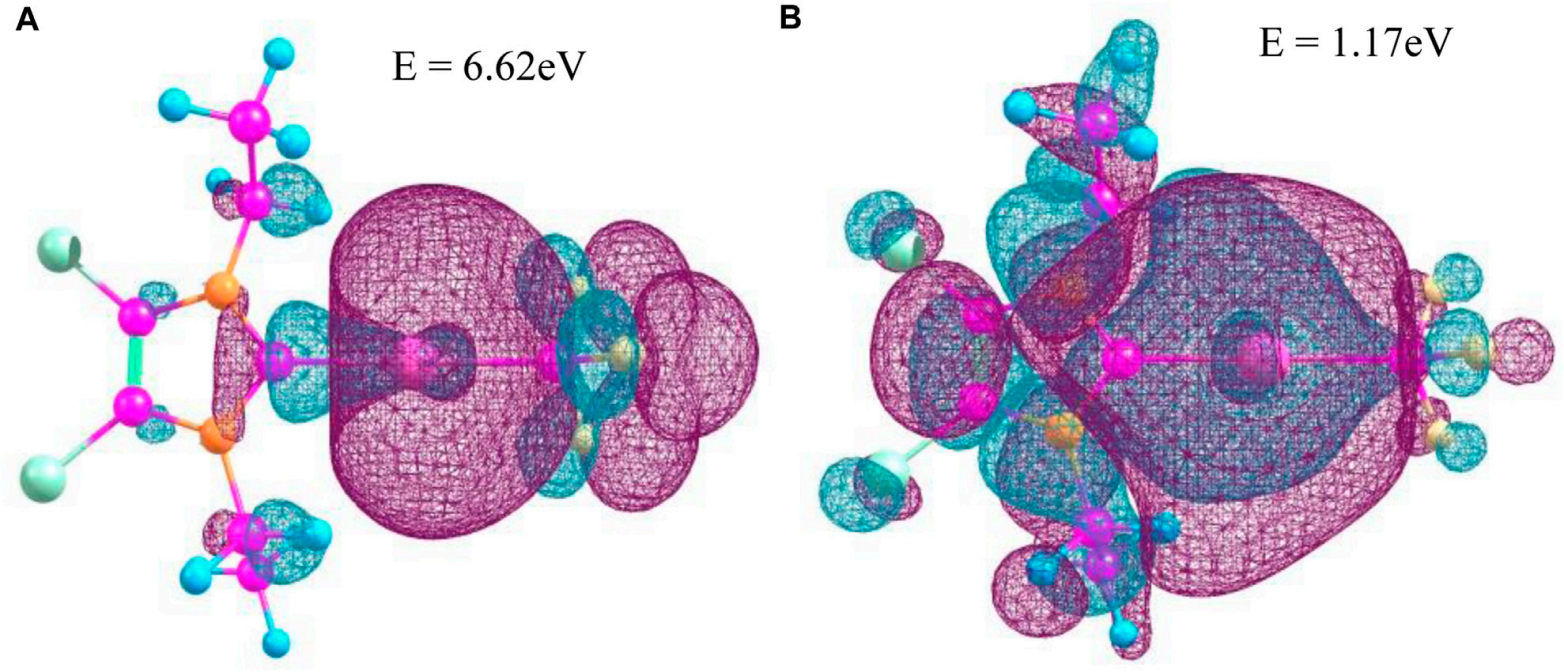
HOMO/LUMO orbitals of 4,5-dichloro-1,3-diethyl-imidazolylidene trifluoromethyl gold(I) (F, yellow; C, pink; H, blue; N, orange; Cl, green; and Au, magenta). **(A)** HOMO (orbital energy 6.62 eV); **(B)** LUMO (orbital energy 1.17 eV).

The characteristics of trifluoromethyl complexes come into a higher bond distance MC–F than C–F, as well as a decrease of F–C–F bond angle and increase in the M–C–F bond angle to the tetrahedral symmetry point group (Wozniak et al., 2019; Péres-Britrián et al., 2017). The bond distance Au–CF_3_ is shorter than Au–CH_3_ and Au–C/Au–Cl, where the Au–Cl bond distance is in the range of ∼2.27 Å (Chang et al., 2015) for [AuCl]^-^. Similar to Au(iii) anion [AuCl_4_]^−^ in scattering processes, vibrational bands have weak Au 2p_3/2_ to 5D transitions, the so called white line, for [AuCl_2_]^-^ ions (Chang et al., 2015), and these weak transitions are the result of a transition from Au 6s/5D hybrid partially occupied to the highest energy level occupied HOMO orbital. For our standard compound, we obtain the HOMO and LUMO orbitals as orbitals 76 and 77, while the total SCF (self-consistent field) density would contain a number of 683 occupied and unoccupied orbitals. Both HOMO and LUMO of 4,5-dichloro–1,3-diethyl–imidazolylidene trifluoromethyl gold(I) are illustrated in Figure 5.

The bond distances calculated using B3LYP/Def2-TZVPP are longer than the free methyl radical bond distances and more imbalanced ranging from 1.087 Å to 2.076 Å. The 3 C atoms of the methyl radical have the bond lengths of 1.089/1.090 Å, equally spaced in all directions with an angle < HCH of 108.1°. The angle characteristics to the methyl radicals in our compound are 106.02° (<HCH) and 112.71° (<CCH). The bond lengths and angles of 4,5-dichloro-1,3-diethyl-imidazolylidene trifluoromethyl gold(I) are presented in Table 2. We report a bond distance for C–Au of 2.065 Å from C1–Au1 and a second bond distance for CF_3_, Au1–C8 of 2.076 Å, the affinity to CF_3_ being higher than to CN_2_ in our case. Lower bond distances of Au–C ligands have been reported by Blaya et al. (2014b) with values declared for the Au–CF_3_ bond of all gold(I) trifluoromethyl complexes to be between 2.031 Å and 2.046 Å. Both the bond distances to CN_2_, C2–N1, and C3–N2 have values of 1.384 Å and angles to the axial plane of 125.3°, while the C4–Cl1 and C3–Cl2 bond distances are 1.702Å set at equally spaced angles of 129°. All C–F bond lengths (Figure 7A; Table 2) from CF_3_ have values of 1.372 Å balanced with an angle of 104.6°, longer than in the free trifluoromethyl radical with distances of the C–F bond of 1.318 Å and an angle of 110.76°, 6.16° lower than in our calculations. The simple ethyl radicals C5–H (from CH_2_ radical) and C5–H (from CH_2_ radical) have bond lengths of 1.087 Å and 1.089 Å, respectively.

**TABLE 2:** Bond lengths of ImEtAuCl_2_CF_3_ optimized at multiple levels of the theory.

Distances in Å												
Basis set/method	Def2-TZVPP			SDD			CEP-121g			QZVP		
Bond *	B3LYP	MP2	HF	B3LYP	MP2	HF	B3LYP	MP2	HP	B3LYP	MP2	HF
Au1–C1	2.065	1.990	2.108	2.052	2.038	2.097	2.066	2.045	2.066	2.064	1.983	2.106
Au1–C8	2.076	2.025	2.098	2.071	2.071	2.105	2.079	2.073	2.079	2.076	2.019	2.099
C1–N1	1.356	1.359	1.335	1.380	1.396	1.356	1.388	1.402	1.388	1.355	1.357	1.335
N1–C2	1.384	1.371	1.378	1.400	1.407	1.393	1.410	1.411	1.410	1.383	1.368	1.378
C2–Cl1	1.702	1.688	1.696	1.771	1.791	1.756	1.781	1.793	1.781	1.701	1.685	1.694
C2–C3	1.357	1.375	1.330	1.370	1.396	1.338	1.376	1.401	1.376	1.357	1.373	1.329
N1–C6	1.469	1.462	1.464	1.485	1.501	1.481	1.496	1.505	1.496	1.469	1.460	1.464
C4–C5	1.523	1.517	1.520	1.538	1.554	1.531	1.545	1.555	1.545	1.523	1.515	1.520
C4–H from CH_2_	1.087	1.087	1.077	1.093	1.100	1.078	1.093	1.098	1.093	1.087	1.087	1.077
C4–H, C5–H from CH_2_	1.089	1.088	1.079	1.095	1.102	1.079	1.095	1.100	1.095	1.088	1.087	1.079
C5–H from CH_3_	1.090	1.088	1.083	1.096	1.104	1.083	1.096	1.102	1.096	1.089	1.087	1.082
C8–F3	1.372	1.366	1.342	1.430	1.454	1.401	1.438	1.458	1.438	1.371	1.364	1.342

The highest values of the Au1–C1 bonds are for the B3LYP/CEP-121 basis set of 2.066 Å, 0.001 Å higher than the calculations at the B3LYP/Def2-TZVPP level of the theory and 0.002 Å higher than the calculations at B3LYP/QZVP and 0.014 Å; the highest discrepancy is obtained using the SDD basis set with the lowest bond length value of 2.052 Å. The 4,5-dichloro–1,3-diethyl–imidazolylidene trifluoromethyl gold(I) compound has the Au–C and C–Au bond lengths 0.4Å higher than the declared value for the Au–C bond in AuIm_2_ (Liu et al., 2013; Benitez et al., 2009) of 1.7 Å–2.06 Å by Liu et al. (2013) and Benitez et al. (2009). The accuracy of MP2 (Møller–Plesset 2 perturbation theory) methods compared to the B3LYP and HF (Hartree-Fock method) ones is very low for very complex molecules containing a high number of atoms or organic parts (peptides and alanine) (Kaminskya et al., 2008). Kaminskya et al. (2008) calculated the error of the MP2 methods with the basis set as being 20–30 kJ/mol in the electronic energy calculations; the values we report for the MP2 with QZVP basis set are the shortest distances Au1–C1 to the Cl_2_-phenyl ring; the calculations and results of Def2-TZVPP have values higher with 0.007 Å of the Au1–C1 distance. All MP2 level calculations for all basis-sets have shorter bond length values and MP2/SDD Au1–C1 has a value of 2.038 Å, while MP2/CEP-121G has a value slightly higher with 2.045 Å, but still lower than B3LYP calculations with the same basis set with values of 2.052 Å and 2.066 Å, respectively.

The chlorine atoms to the phenyl ring bond lengths range from 1.685 Å calculated at MP2/QZVP to 1.793 Å at MP2/CEP-121G, both Cl atoms being placed symmetrically at 129.22° to the C2 and C3 atoms of the phenyl ring. Overall, the structure is balanced at the central symmetry plane formed by two C atoms and Au, C1–Au1–C8, while 110.83⁰ to C (in CH_2_) and 90⁰ to the symmetry plane is obtained for the two ethyl–methyl groups.

## XRD measurements of 4,5-dichloro-1,3-diethyl-imidazolylidene trifluoromethyl gold(I) for nanomaterial characterization

### Crystal structure analysis

**FIGURE 6:**
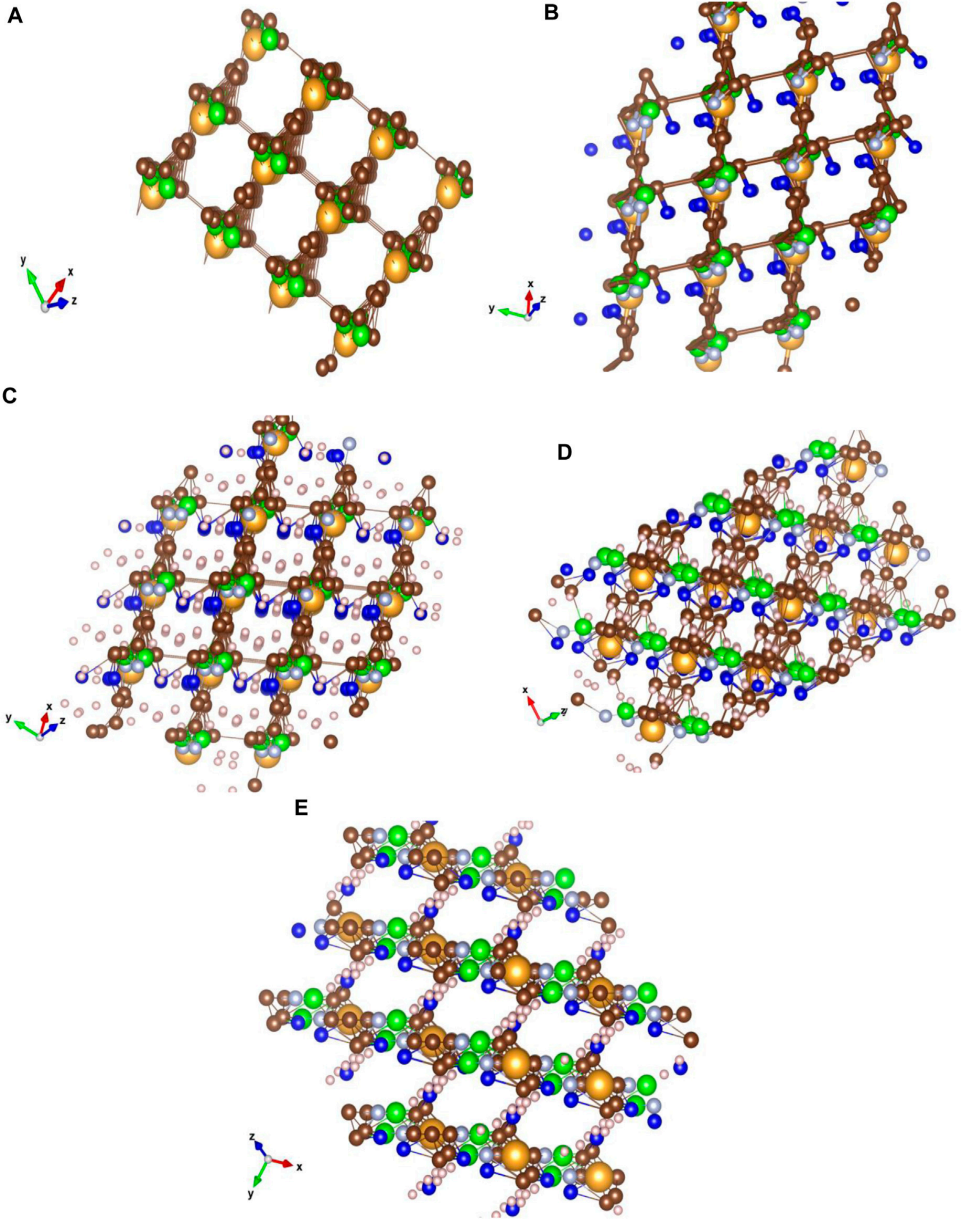
Crystal structure simulations of 4,5-dichloro-1,3-diethyl-imidazolylidene trifluoromethyl gold(I) for nanomaterial characterization. Characteristic to the ImEtAuCl_2_CF_3_ crystal matrix is the bonding at 60° of the carbon basket surrounding the Au atom to the next C-ring structure through a fluorine-hydrogenated C bond, while nitrogen would have a higher noble metal affinity bonding to the Au–C structure forming AuNCl^−^ sites with high electron affinity compared to the C lattice surrounding the Au atoms. The structure was optimized using Gaussian 16 and Avogadro software and analyzed using the VESTA software (Cl, green; F, dark blue; N, light blue; H, white; C, brown; and Au, yellow). **(A)** Crystal structure of ImEtAuCl_2_CF_3_ optimized at the B3LYP/Def2-TZVPP level of the theory with only Au, Cl, and C atoms and characteristic bond lengths of the Au–C bond from 0 to 2.065 Å and C–C between 0 and 1.358 Å, forming a carbonaceous matrix that surrounds the Au and Cl atoms in a square matrix; **(B)** the same type of matrix with similar bond lengths of Au–C and C–C with the addition of C–N and C–F bonds to the carbonaceous matrix; no bond between F and C is observed. The F atom places itself in the middle of the matrix next to the Au atom; H bond and atoms were removed for clarity; **(C)** YXZ view of the crystal with focus on the fluorine-hydrogenated bonds between the molecules; **(D)** XYZ view with focus on the carbon matrix of the crystal; **(E)** ZXY view of the crystal lattice with focus on the carbon rings and the fluorine-hydrogenated bonds. The Cl^−^ atoms and N atoms gather around the Au atoms creating a basket, while the F^−^ atoms were involved in creating a bond at 90° with the next molecules in a homogenous structure. The crystal lattice was built using 2 × 2 × 2 (a × b × c) Miller indices with a lattice unit cell of 4.275 Å on X and Y and 80 Å on Z at an angle of 60⁰ on *z*-axis and 90⁰ on *x*-axis and *y*-axis. The crystal matrix on all the planes has an inhomogeneous structure, the exceptions being YXZ at 20⁰, XYZ at −10⁰ to −70⁰ and ZXY at −10⁰ planes, where a coherent pattern can be seen between all molecules. Characteristic to the Au-fluorinated carbonaceous matrix is the presence of the Au atom in the center of a carbon ring bonded with a fluorine-hydrogenated carbonaceous bond with other layer molecules. The chlorine and nitrogen atoms follow Au closely through very strong interatomic forces. The lattice allows the presence of four molecules into a unit cell at 60⁰ plane angle.

The ImEtAuCl_2_CF_3_ crystal simulation was performed in the Vesta software on a pre-optimized molecular structure in Gaussian 16 at the B3LYP/Def2-TZVPP level of the theory using density functional theory calculations with full orbital populations and triple-ζ frequency calculations (Figures 6A–E). A number of bond lengths and the unit cell have been optimized by trial and error to obtain the slab of the crystal with different γ-orientations of the crystal planes.

### Single-crystal XRD structure

**FIGURE 7:**
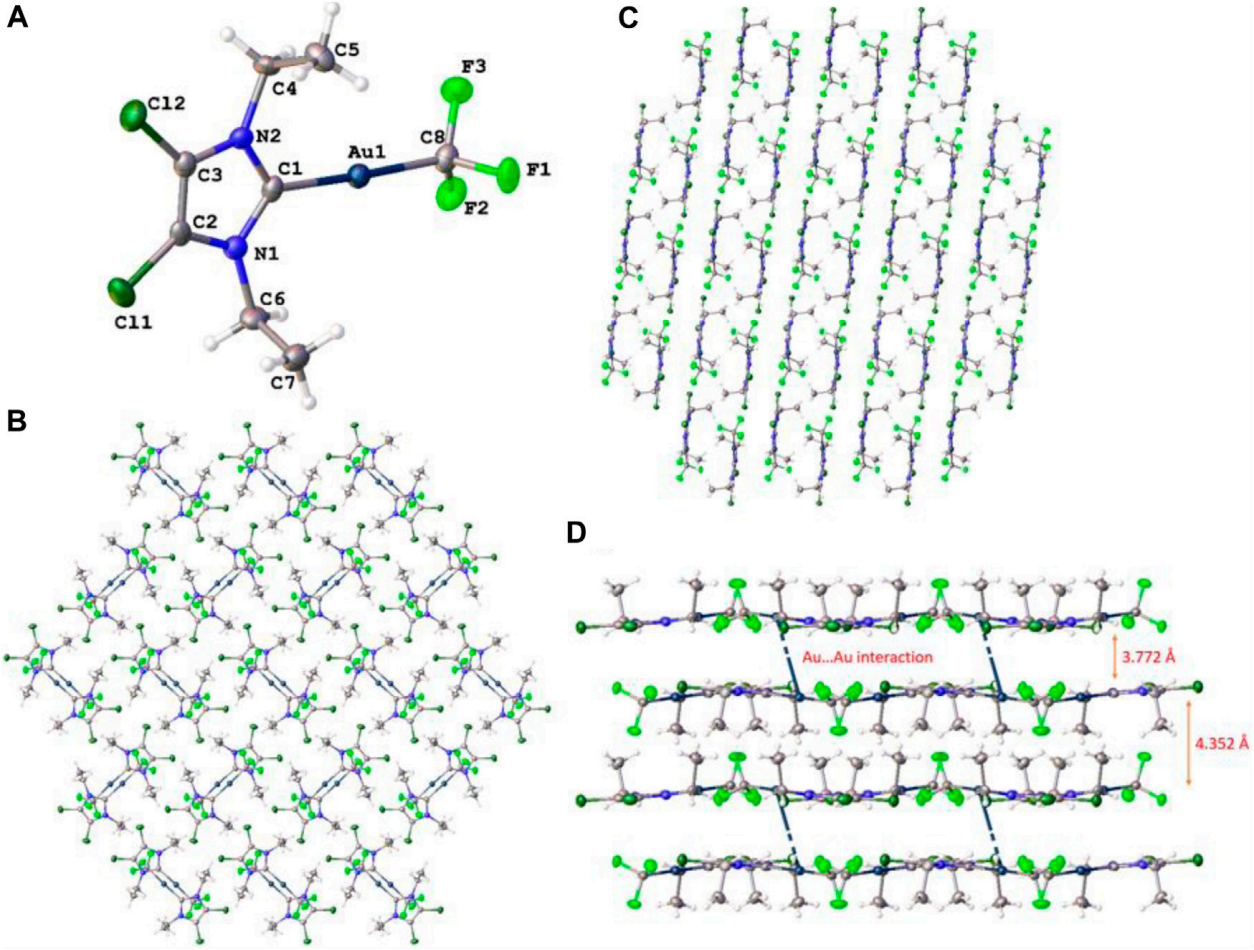
**(A)** Crystal structure of the 4,5-dichloro-1,3-diethyl-imidazolylidene trifluoromethyl gold(I) complex; ellipsoids have been drawn at 50% probability. Packing and interlayer distance **(B)** viewed along the *a*-axis, **(C)** *b*-axis, and **(D)** *c*-axis has been shown.

The crystals belong to the monoclinic space group P2_1/c_ (*β* = 98.188°, V = 1219.69 Å^2^), and the unit cell is composed of a very weak aurophilic dimer with rather longer Au–Au short contact distance (3.772 Å), compared to the previously reported similar dimeric carbene–Au(I)–CF_3_ complexes (Blaya et al., 2014b). The coordination geometry of the Au(I) complex is linear (angle C1–Au(I)–CF_3_ = 176.93°) and slightly distorted with a tad bit shorter Au–CF_3_ distance (d_Au–CF3_ = 2.029 Å) compared to previously reported distances found in Au(I) trifluoromethyl complexes containing carbene, isonitrile, or C_6_F_5_ ligands (d_Au–CF3_ = 2.031–2.046 Å) (Blaya et al., 2014b). The molecule has been found to exist in antiparallel conformation with another monomer so as to facilitate closer distance between two Au centers, while minimizing steric repulsion between CF_3_ and carbene center, as shown in Figure 7B. It is noteworthy to mention that the two methyl groups attached to carbene NCH_2_ moiety of a molecule exist in the cis configuration to each other, thereby allowing a close approach between two Au centers and widening the distance from the adjacent molecule in the next row. Detailed list of bond angles and bond distances have been summarized in Table 3 taking into account van der Waals forces and short contact angles.

**TABLE 3:** List of bond lengths, bond angles, torsional angles, van der Waals interactions, and short contact obtained from single crystal XRD structural analysis.

**Type of bond**	Bond length(A)	Type of angle	Bond angle ()
Au1–C8	2.029	C1–Au1–C8	176.936
C1–Au1	2.024	Cl1–C2–C3–Cl2	1.166
C1–N2	1.345	C3–N2–C4–C5	88.293
C1–N1	1.348	C2–N1–C6–C7	−79.407
C2–N1	1.395	van der Waals interactions	
C3–N2	1.378	Participating atoms	Distance(A)
C3–C2	1.351	Au1–H2B	3.377
C2–Cl1	1.679	Cl1–H4B	2.814
C3–Cl2	1.696	H5C–H5C	2.201
C8–F1/F2/F3	1.373/1.371/1.368	F2–H4A	2.468
N2–C4	1.469	Short contact	
N1–C6	1.463	Au(l)–Au(l)	3.772

Detailed investigation of the packing of the crystal structure (Figures 7B–D) revealed the presence of various weak non-covalent interactions that exist between adjacent rows of molecules. Apart from aurophilic interactions, each molecule engages with four surrounding molecules via four different van der Waals forces, which are summarized in Table 3. Among these interactions, each molecule engages with two adjacent molecules in the same row via F2···H4A and Cl1···H4B van der Waals forces and two molecules in the next row via Au1···H5B and H5C···H5C interactions. Interestingly, one Au center engages with another nearby Au center via well-known aurophilic interaction (short contact), albeit weak and with a methylene proton of another molecule, positioned in the opposite row to the first one, thereby resulting in different interlayer distances. The interlayer distances have been shown in Figure 7C, where the packing has been viewed along the *c*-axis.

### X-ray powder diffraction data (XRD)

A set of eight experiments at different temperatures (0–298 K) and pressures have been run using the powder X-ray diffraction method (Doumeng et al., 2021; Khan et al., 2020; Zhou et al., 2018) to determine the crystallinity and structure of the sample. For the characterization of nanomaterials and deposited complexes at the nanoscale, combinations of tools such as TEM (transmission electron microscopy), EXAFS (extended X-ray absorption fine structure), and XRD (Holder and Schaak, 2019; Gao and Lowry, 2018) are run to obtain particle size distributions and interlayer plane distances using TEM for the localized nanostructure size and powder XRD for an average nanostructure size. Further measurements can be carried out for structural characterization of the as-deposited nanomaterials using synchrotron radiation (X-ray absorption near edge structure (XANES) and X-ray small angle scattering (XSAS)) (Gao and Lowry, 2018; Amidani et al., 2021).

**FIGURE 8:**
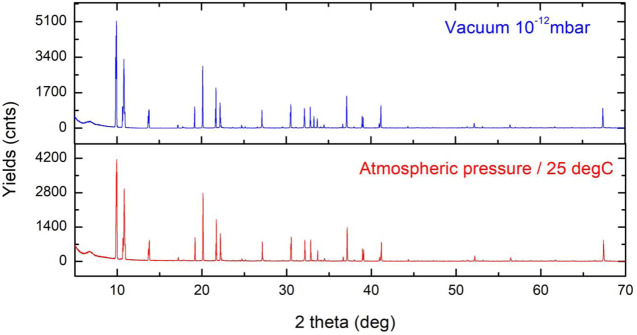
XRD of the Au compound under vacuum condition (10^−12^mbar) and at room temperature and atmospheric pressure. The two graphs show very small differences under two different conditions, and the highest changes are observed between 30 and 35 2 θ(°).

Two measurements have been performed to verify the stability and induced chemistry of the ligands of the compound (Figure 8) when exposed to air. Both NMR and XRD equipment work by having the sample handled in air (atmospheric pressure and room temperature). Its stability is an important characteristic of an FEBID precursor. The measurement in vacuum at 10^−12^mbar compared to the atmospheric pressure and 25 °C presents sharper peaks with reduced noise and a reduced width of the peaks.

At 2θ (°) 30–35 °, we observe the presence of a third peak corresponding to the Au–CF_3_ bond increasing the bond distance with the change of pressure. With change in the pressure and temperature, CF_3_ as the most volatile radical of the compound changes the bond length in the range of 10^–1^ Å.

**FIGURE 9:**
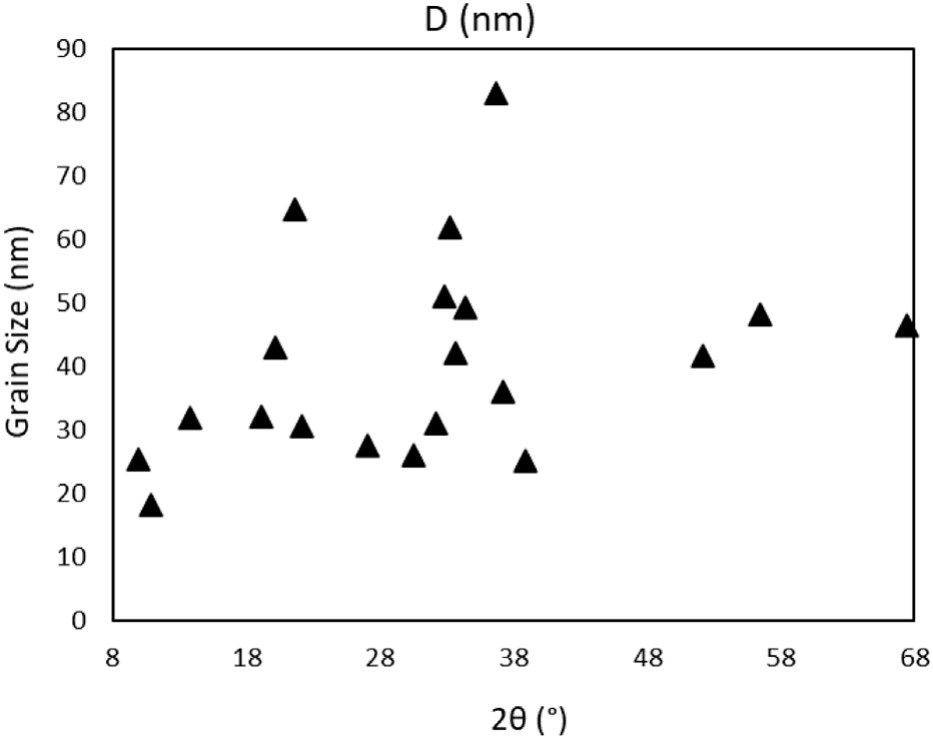
Powder grain size distribution of the Au compound.

The size of 4,5-dichloro-1,3-diethyl-imidazolylidene trifluoromethyl gold(I) grains (Figure 9) is calculated using Formula (1), where *λ* = 1,54060 Å and *β* is the FWHM width of the peak in radians and θ position of the peaks/2 in radians:

$$D_{p}=0.9*\lambda /\;\beta \cos\theta .$$ (1)

The highest grain size batch is of 83 nm at 0.64 rad θ, with an average grain size of all peak batch of 41 nm. Further analysis of XRD powder data of the compound gives information on the crystallinity by using the position of the 2θ peaks on the XRD spectrum, the spectrum recorded under the vacuum was used as cleaner and without H_2_O presence. The crystallinity of the precursor is calculated using Eq. 2 (Zhong et al., 2009) with a value of 54.427%:

$$Crystallinity=Area\;of\;crystalline\;peaks\;*\;100\;/\;Area\;of\;all\;peaks\;\left(crystalline+amorphous\right).$$ (2)

A crystallinity of up to 55% is expected, with the grain size limited to an average of 41 nm, and the highest grain of 82.86 nm, rather small compared to a grown crystal structure or multiple grown crystals in the powder structure. The complex is in the amorphous phase mixed with small grains in the form of nanostructures.

**FIGURE 10:**
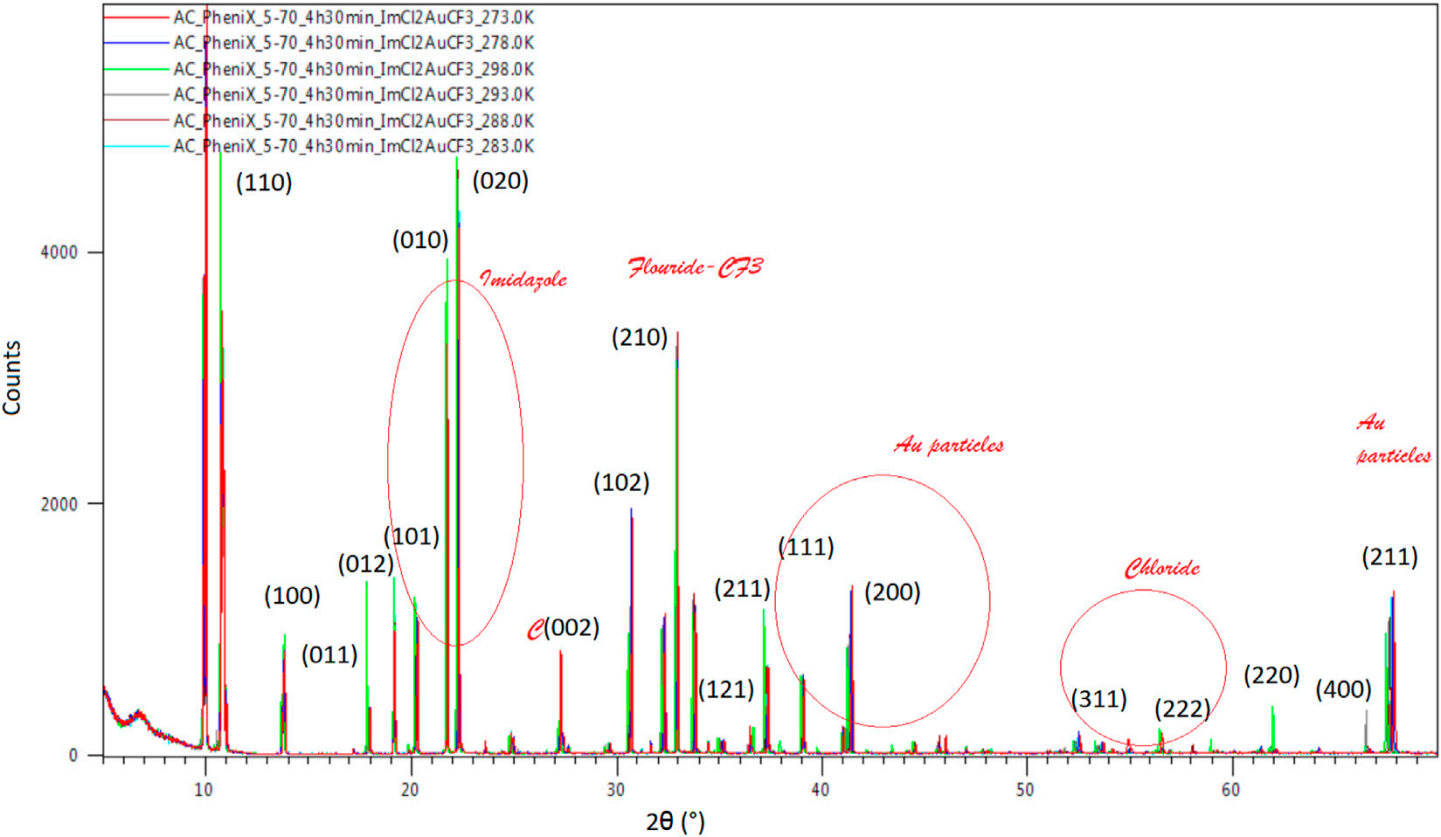
Temperature dependence of XRD data of the Au compound to 2θ (°). A normalized behavior with increasing amplitude and constant delay is observed by changing the temperature from 273 to 298K.

An increase in the peak amplitude is observed for the 298 K spectrum compared to the rest, a behavior expected at RT, while a separate increase in the singular peak amplitude is observed for 273K and 278K at 2θ(°) 26 corresponding to the C (002) phase, 298K at 2θ(°) 62 corresponding to Au–chloride (220) body-centered cubic (bcc) plane of the crystalline powder at 293 K at 2θ(°) 66.5 corresponding to the (400) bcc Au–chloride plane and phase (Figure 10). A separate view of the planes and phases of the crystalline powder and temperature is illustrated in Figure 10, where phases of bcc are combined with face-centered cubic (fcc), and an intermediate highly hydrogenated layer creating the interspacing of the atom and network positioned between fcc and bcc is observed, Au surrounding itself with chlorine atoms.

## Suitability of FEBID of the precursor

### Negative ions of 4,5-dichloro-1,3-diethyl-imidazolylidene trifluoromethyl gold(I)

**FIGURE 11:**
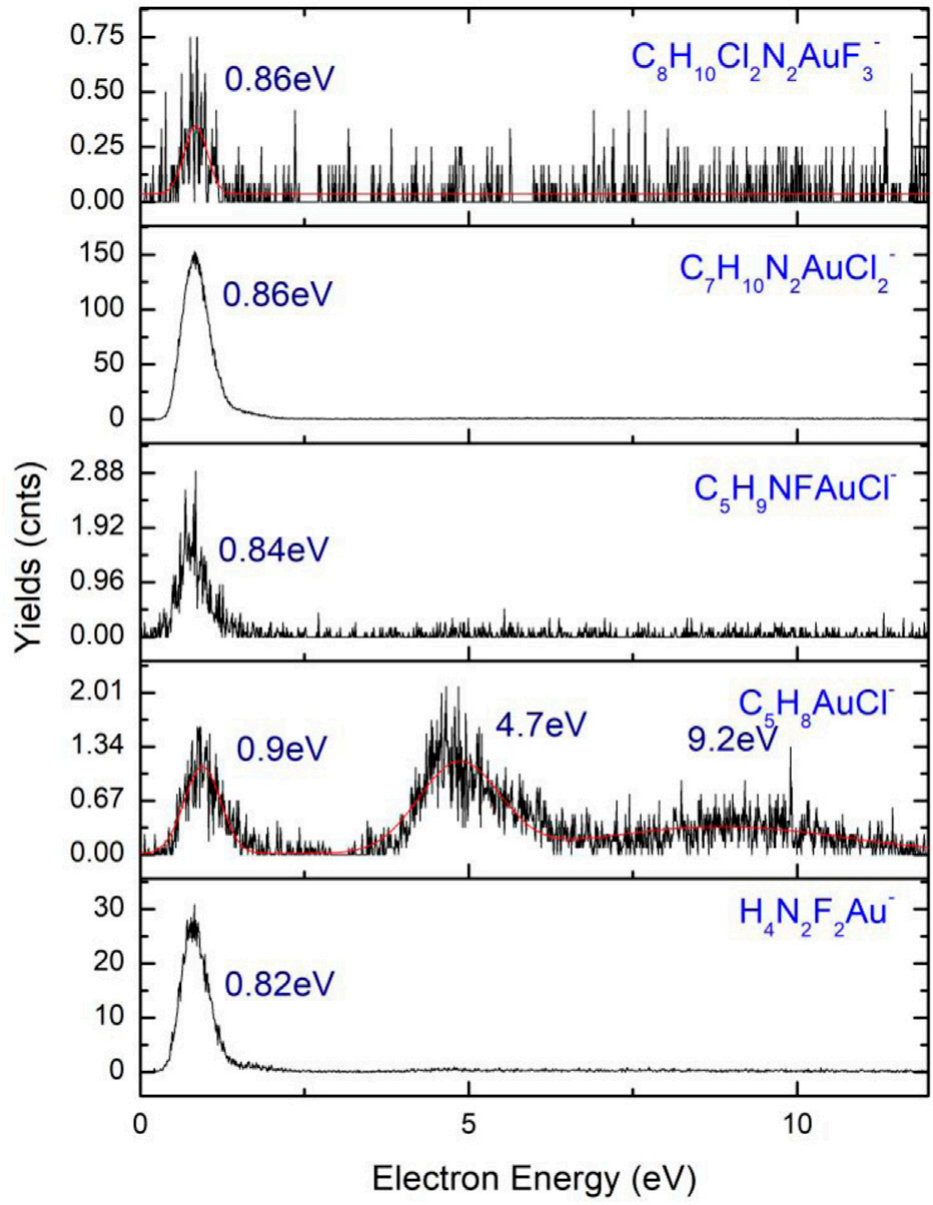
Anions of 4,5-dichloro-1,3-diethyl-imidazolylidene trifluoromethyl gold(I) *m/z* 267 (H_4_N_2_F_2_Au^−^) to *m/z* 458 (C_8_H_10_Cl_2_N_2_AuF_3_^−^).

A number of six gold(I)-containing ions (Figure 11) are the results of fragmentation of 4,5-dichloro-1,3-diethyl-imidazolylidene trifluoromethyl gold(I), as the only anion that contains a metal atom, found in the dissociative electron attachment (DEA) process (Shuman et al., 2011; Sauer et al., 2002) of the compound. The two higher mass fragments, namely, C_5_H_10_N_2_F_2_AuCl_2_^−^ (*m/z* 389) and C_7_H_10_N_2_FAuCl_2_^−^ (*m/z* 334), are rather noisy with low cross sections, both having one resonance peak with the highest value before 1 eV. With higher cross-section values, the C_7_H_10_N_2_AuCl_2_^−^ anion has an average count value of 150 counts at the maximum of the resonance, falling at 0.86 eV, with an average width of the resonance peak at 2.03 eV. A lower value of the cross-section with maximum counts of two counts characterizes the anion C_5_H_9_NFAuCl^−^ (*m/z* 334), presenting similar shape to the higher mass anion at *m/z* 389. The electron energy characteristic to the resonance of the anion (*m/z* 334) is 0.84 eV, with a width of 1.81 eV. In the normal dissociation process of C_8_H_10_Cl_2_N_2_AuF_3_, the parent is excited by the collision with an electron and dissociates into an anion and a neutral fragment, but at kinetic energies lower than 0.1 eV, a process of thermal decomposition is the path followed in the dissociation of the precursor, a process observed in the formation of C_7_H_10_N_2_FAuCl_2_^−^ at kinetic energies as low as 0.03 eV. A transition from the π HOMO state to LUMO in π* takes place at the resonance incident energy for a bond dissociation energy of 0.89 eV with the loss of CF_2_ in the neutral state.

At *m/z* 300, characterized by a very low cross-section value, the C_5_H_8_AuCl^−^ anion is found. The three peak resonances of the C_5_H_8_AuCl^−^ anion are at electron energies of 0.9 eV, 4.7 eV, and 9.2 eV, with high characteristic widths and a noisier shape. The widths of the three resonances are in the range of ∼2–3 eV, with values of 1.53 eV (0.9 eV), 3.38 eV (4.7 eV), and 3.3 eV (9.2 eV). The smaller mass anion of the six gold(I)-containing ions is H_4_N_2_F_2_Au^−^ characterized by the presence of nitrogen and fluoride atoms in its composition and a high cross-section. The peak of the highest resonance is found at 0.82 eV with a maximum of the peak of 32 counts and a width of 1.67 eV. The smaller amplitude resonance falls at 4.6 eV having a width of 8 eV and a number of counts lower than two counts. Another Au-containing anion is H_4_N_2_F_2_Au^−^ with its resonance peaking at 0.82 eV characterized by a peak width of 0.18 eV. The bond dissociation energy of the ion formation has a value of 0.021 eV with a maximum kinetic energy of 3. 75 eV. A π to σ* transition is characterized by the HOMO to LUMO transition in the dissociation process for a C_2v_ symmetry of the formed H_4_N_2_F_2_Au^−^ anion.

The parent anion C_8_H_10_Cl_2_N_2_AuF_3_^−^ is present with a resonance peaking at 0.86 eV, showing a very low cross-section with a reduced number of counts (<0.75 counts). At the excitation of the C_8_H_10_Cl_2_N_2_AuF_3_, a temporary negative ion is formed with a maximum kinetic energy of 1.14 eV, where conservation of the C_2v_ symmetry state is observed for the excited parent anion in the transition from π to π*.

**FIGURE 12:**
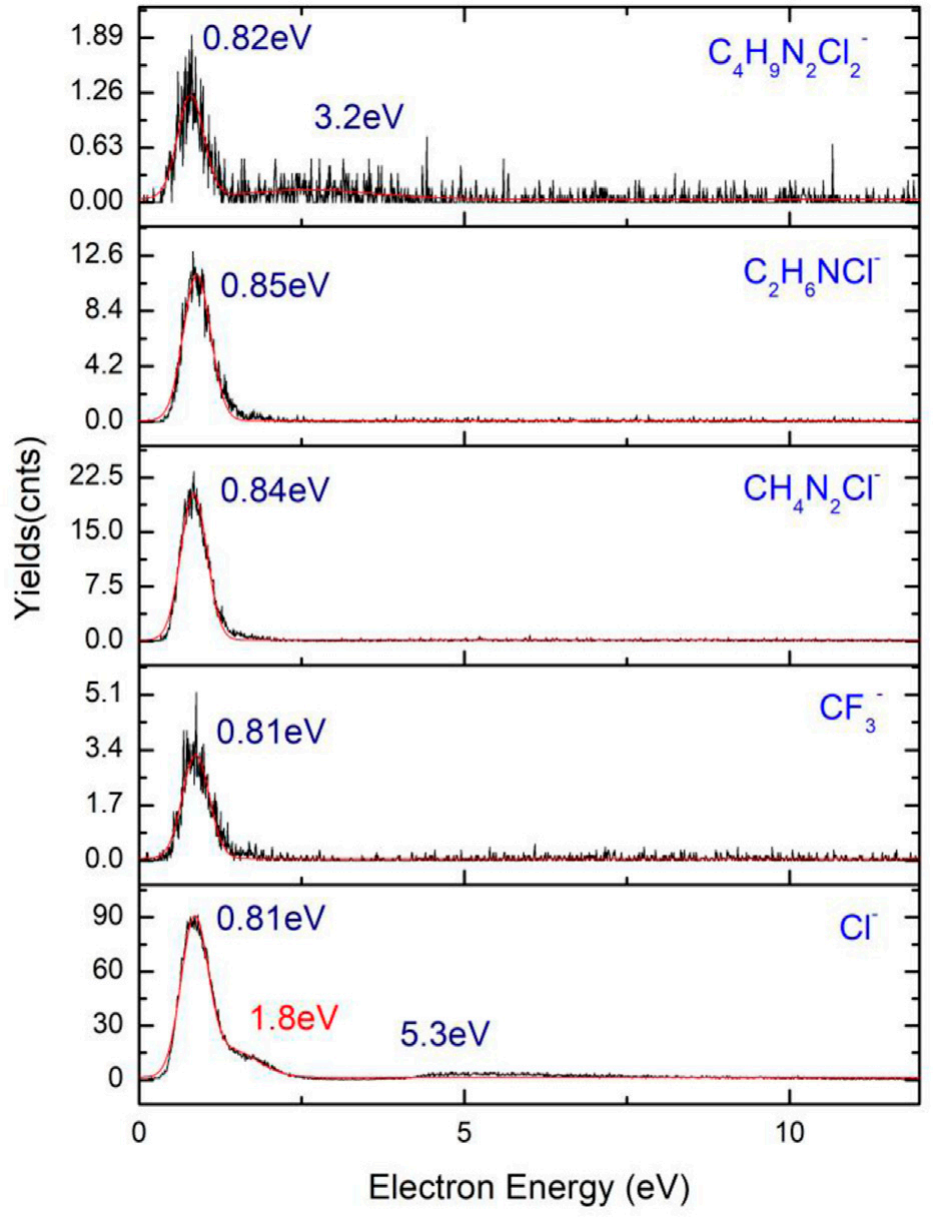
Organic parts and volatile fragment anions of 4,5-dichloro-1,3-diethyl-imidazolylidene trifluoromethyl gold(I).

An equal number of organic anions (Figure 12) are found as a result of DEA fragmentation of the precursor, the most abundant being Cl^−^ and CH_4_N_2_Cl^−^. These five lower mass fragments are particularly interesting because of the lack of any metal atom in their composition, depositing, and releasing as a result of collision and ionization in the interaction with a secondary electron having only volatile fragments and organic materials, increasing the level of contamination of the FEBID structures. Another very abundant anion is the C_2_H_6_NCl^−^ ion having only one resonance peak at 0.84 eV with a width of 1.7 eV and 24 counts and a relatively high cross-section compared to the rest of the ions presenting values under 10 counts.

The Cl^−^ ion corresponding to an *m/z* of 35 presents a resonance peak at 0.85 eV with high cross-section values, being the most abundant anion result of the fragmentation of the precursor. A shoulder corresponding to the same resonance is observed at 1.8 eV, while a wider resonance peak with a width of almost 4 eV is observed at 5.3 eV. The formation of the Cl^−^ ion is a transition from the π to σ* process characterized by a bond dissociation energy (BDE) of 0.59 eV. A maximum of the kinetic energy of the Cl anion is obtained with a value of 0.26 eV. Fragmentation of 4,5-dichloro-1,3-diethyl-imidazolylidene trifluoromethyl gold(I) follows the steps in relation (1) to the result of a chloride anion and of a higher mass neutral fragment, C_8_H_10_Cl_2_N_2_AuF_3_ + e^−^ → C_8_H_10_Cl_2_N_2_AuF_3_^−^ → C_8_H_10_ClN_2_AuF_3_ + Cl^−^ (1). Xuan et al. (2014) presents the fragmentation of 1,2-dichlorobenzene at low energy DEA studies by ion mass spectroscopy, time-of-flight, and VMI on the fragmentation of the compound, resulting in a Cl^−^ ion with two resonances, 1.2 eV and 6 eV, the latter being a wider resonance of the anion possibly corresponding to the two isotopes of chloride, namely, ^35^Cl^−^ and ^37^Cl^−^.

The chloride anion is not an atypical ion in the fragmentation process of the compounds containing Cl, a majority of them forming the chloride anion as a product of reaction in the induced chemistry during the interaction of the molecule with electrons. In the fragmentation of diatomic molecules, 1,2-dichlorobenzene (1,2 - C_6_H_4_Cl_2_) undergoes a transition from σ to σ* that further initiates the fragmentation of the molecule with the resulting fragments being C_6_H_4_–Cl+Cl^−^, with the chloride anion in the ^1^∑_g_* excited state and O_h_ geometry. A study of four chlorine-containing compounds (CCl_4_, CH_2_Cl_2_, CH_3_Cl, and CHCl_3_) (Scheunemann et al., 1980) at DEA fragmentation exhibits the presence of the chloride anion at energies close to 0 eV. The positions of the resonances of the four anions are presented in Table 4. Each of the ion has the highest amplitude peak close to 0 eV at an electron energy of 0.0 ± 0.05 eV, and the second resonance peak between 6 eV and 8 eV. Cl^−^ from CCl_4_ has a shoulder of the first resonance of chloride falling at 0.75 ± 0.05 eV, with a value of the bond dissociation energy of C–Cl ligand of 3.3 ± 0.3 eV and a characteristic electron affinity of EA (Cl_2_) = 2.35 ± 0.1 eV. The electron affinity reported by ^*NIST Database*^ is in good agreement with the values of 2.5 ± 0.2 eV reported by Scheunemann et al. (1980).

**TABLE 4:** Electron affinities of ions including C_7_H_10_N_2_AuCl_2_^−^(*m/z* 389), H_4_N_2_F_2_Au^−^(m/z 267), C_2_H_6_NCl^−^ (*m/z* 79), and CF_3_^−^ (*m/z* 69).

Ions	C_7_H_10_N_2_AuCl_2_^−^	H_4_N_2_F_2_Au^−^	C_2_H_6_NCl^−^	CF_3_^−^
**VEA(eV)**	0.506	1.090	0.561	2.820 (Richardson et al., 1975) *VDE value
**EA(eV)**	3.0327	3.9009	2.9781	3.3157

The two lower value cross-section fragments, namely, CF_3_^−^ and C_4_H_9_N_2_Cl_2_^−^ lacking the presence of any metal atom, have amplitudes in the range of ∼2–5 counts, with the highest amplitude peak for C_4_H_9_N_2_Cl_2_^−^ falling at 0.82 eV having a width of the resonance of 1.26 eV, while the second peak of the same resonance has its maximum at 3.2 eV with the width of the peak of 2.16 eV. The CF_3_^−^ anion has its maximum amplitude of the first resonance peak at 0.81 eV with a width of 1.5 eV and the second peak maximum at 7.2 eV characterized by a width of 6.6 eV. BDE of the Cl^−^ formation is 0.59 eV and has a specific kinetic energy of 0.26 eV, where the HOMO to LUMO transition is from π to σ* with the anion having C_s_ symmetry.

Manaa (2017) defines the value of the calculated electron affinity from Gaussian 4 simulations at the CCSD(T) (coupled cluster single-double and perturbative triple) level of the theory as the sum of the values of energy E_e_ for the neutral and the anion with added zero-point corrections of the two values. (2) EA = [E_e_ (optimized neutral) + ZPE (neutral)]–[E_e_ (anion) + ZPE (anion)]. A similar relation is used for the cation ionization potential at ZPE (zero-point energy); (3) IP = [E_e_ (cation) + ZPE (cation)]–[E_e_ (optimized neutral) + ZPE (optimized neutral)]. In the dissociative electron attachment calculations of VEA and EA run at the DFT level, the values of the transitions from HOMO to LUMO orbitals are related to excitation energies with the formation of a temporary negative ion (TNI). The electron affinity and electronegative potential (absolute electronegativity or absolute hardness) of the anion results of the DEA process of 4,5-dichloro–1,3-diethyl-imidazolylidene trifluoromethyl gold(I) follow (2) with resulting values in the range of 2.9–3.9 eV (see Table 4). The electron affinities (EA) of the products have been calculated at room temperature (298.15 K), the zero-point energy (ZPE) corrections being highly sensitive to the input temperature. The highest value of the electron affinity is obtained for mass *m/z* 267 corresponding to H_4_N_2_F_2_Au^−^ with an EA value of 3.9 eV (∼0.143357 Hartree). Lower electron affinity values are calculated for the Cl^−^ ion and C_7_H_10_N_2_AuCl_2_^−^ with values of 3.32 eV (∼0.12185 Hartree) and 3.03 eV (∼0.11145 Hartree), respectively. The VEA (vertical electron affinities) values from our Gaussian 16 simulations at the B3LYP/LANL2DZ level of the theory are calculated using the natural bond orbital populations (NBO) and pole p3+ calculations (Table 4 **)**. The vertical electron affinities (VEA) (Jiao et al., 2016; Li et al., 2002) are calculated for each ion with high cross-sectional values ranging from 0.51 eV to 1.09 eV. VEA of 4,5-dichloro-1,3-diethyl-imidazolylidene trifluoromethyl gold(I) has a value of 0.24 eV, 0.15 eV higher than the excited anion parent with a value of VEA of 0.096 eV, similar to other compounds containing C–H bonds; example of CH_3_^−^, SiH_3_^−^, and CHCH_2_^−^ (Amati et al., 2020). The anion parent could not be determined experimentally as it is characterized by a short-lived life and instability in the ^2^A_1_’ state.

In the fragmentation of 4,5-dichloro-1,3-diethyl-imidazolylidene trifluoromethyl gold(I), multiple pathways are possible, described by relations (4) and (5), with the formation of the CF_3_^−^ (*m/z* 69) anion with C_7_H_10_Cl_2_N_2_Au as the neutral fragment, while the second fragmentation pathway results in the formation of the C_7_H_10_N_2_Cl_2_Au^−^ (*m/z* 389) anion and CF_3_ as a neutral fragment: C_8_H_10_N_2_Cl_2_AuF_3_ + e^−^ → C_8_H_10_Cl_2_N_2_AuF_3_^−^ → C_7_H_10_N_2_Cl_2_Au^−^ + CF_3_ (4) and C_8_H_10_N_2_Cl_2_AuF_3_ + e^−^ → C_8_H_10_Cl_2_N_2_AuF_3_^−^ →C_7_H_10_N_2_Cl_2_Au + CF_3_^−^ (5). While CF_3_ in its ground state, ^2^A_1_ has a C_3v_ geometry; for the CF_3_^−^ anion, the symmetry point group conserves (C_3v_), but the excited state of the anion transitions to ^1^A_1_ presenting two peaks of the resonance, namely, at 0.81 eV and 7.2 eV; other higher excited states of the anion correspond to E″_1_ and E″_2_. BDE of the CF_3_^−^ formation is 0.53 eV with a maximum kinetic energy of 0.47 eV. The CF_3_^−^ anion from 4,5-dichloro-1,3-diethyl-imidazolylidene trifluoromethyl gold(I) is observed to behave similarly to CF_3_^−^ in 5-trifluoromethanesulfonyl-uracil (Ameixa et al., 2018; Bald et al., 2007), which is used extensively in the research of cancer therapy as a potential radiosensitizer, reducing the amount of radiation needed for the treatment of the cancer cells. Defined by an electron affinity (EA) of 1.69 eV, the fragmentation of 5-trifluoromethanesulfonyl-uracil (OTfU) (Ameixa et al., 2018) in a neutral fragment and CF_3_^−^ takes place at an electron energy characterized by four resonance peaks, namely, at 0.01 eV, 2.35 eV, 4.75 eV, and 8.45 eV, which is the result of dissociation of the S–CF_3_ ligand. The CF_3_^−^ ion in the hexafluoroacetone azine ((CF_3_)_2_C = NN = C(CF_3_)_2_) (Bald et al., 2007) reaction has its resonances falling at higher energies representing typically a bond cleavage with the fragmentation of a C–CF_3_^−^, a C bonded to CF_3_ having a lower bond dissociation energy than the Au–CF_3_ bond cleavage for 4,5-dichloro-1,3-diethyl-imidazolylidene trifluoromethyl gold(I). Values of 1.61 eV are reported for the affinity of CF_3_^−^ (Bald et al., 2007) with the DEA resonance peaking at two energies, namely, 3.8 eV with the highest amplitude and 7.3 eV resonance with a lower amplitude, with less than <10 counts. Calculations of the vertical electron affinities (VEA) and bond dissociation energies show higher bond strength of the Au–CF_3_ ligand compared to the organic ligands in ImCl_2_EtCF_3_Au.

### Proton NMR stability measurements

The proton NMR measurements and water NMR measurements are rather simple measurements used often in the pharmacology industry for stability analysis of complex drugs and compounds and the degradation study of these compounds in specified conditions (pressure, temperature, and luminosity). The applications of the proton NMR studies are not limited to only stability analysis but also have implications in the study of the structure and bonding of complex compounds to certain proteins (RNA signal assignment and validation (Barton et al., 2013), probing metallic-aromaticity (Bass et al., 2020), or structural changes (n-membrane lactones isomerism (Marell et al., 2014). The most common example of the use of this type of measurement is the proton NMR studies to proteins (Taraban et al., 2017) in different storage, transportation, and daily-use conditions. The proton NMR and water NMR offered in these circumstances provide a comprehensive view on the stability of the compound and the time it takes for the chemical complex to degrade or to form new bonds as a result of transition processes to a new form or a chemical reaction induced by temperature or changes in the environmental conditions (pressure or light).

**FIGURE 13:**
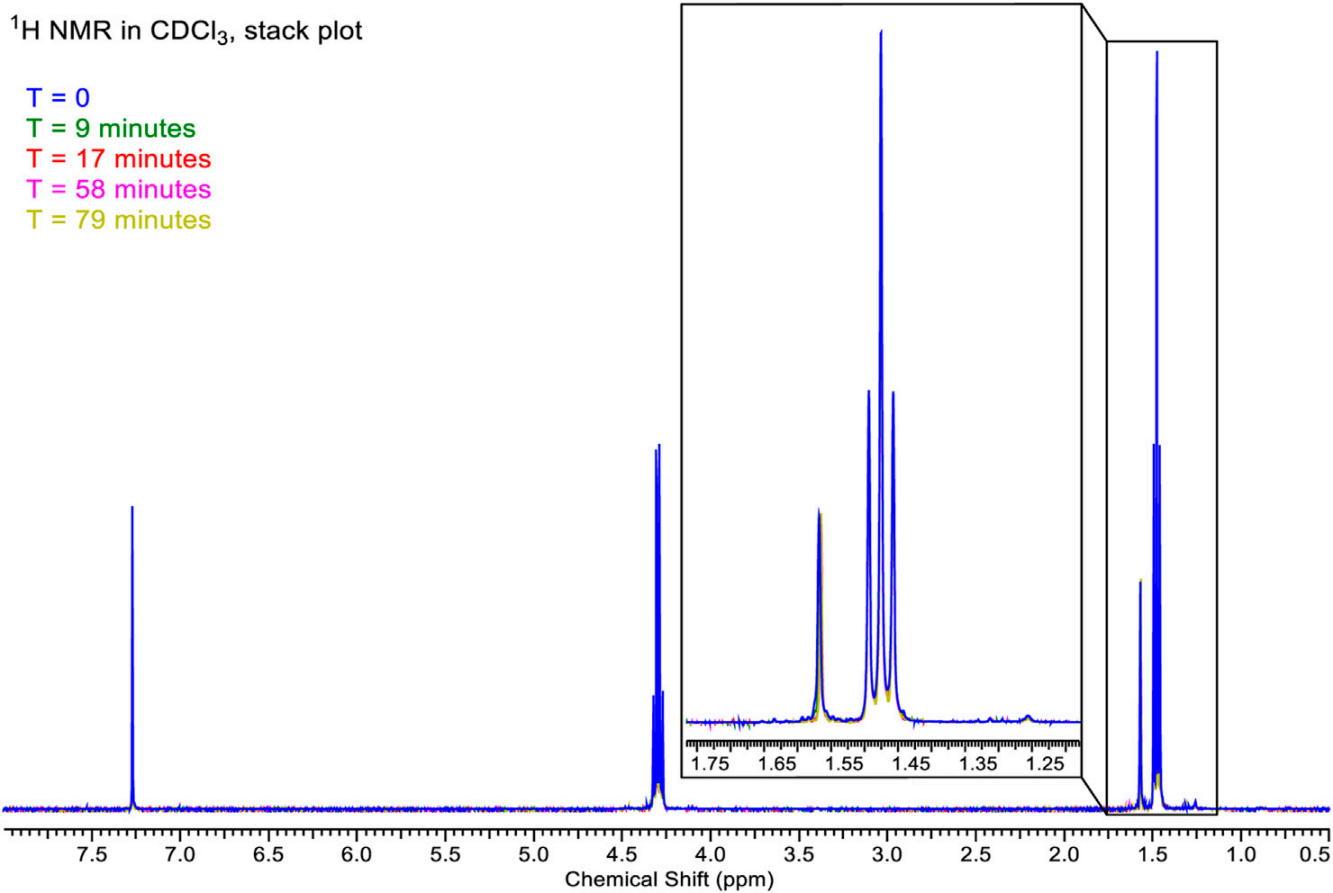
Chemical shift of 4,5-dichloro-1,3-diethyl-imidazolylidene trifluoromethyl gold(I) in CDCl_3_ solution.

**FIGURE 14:**
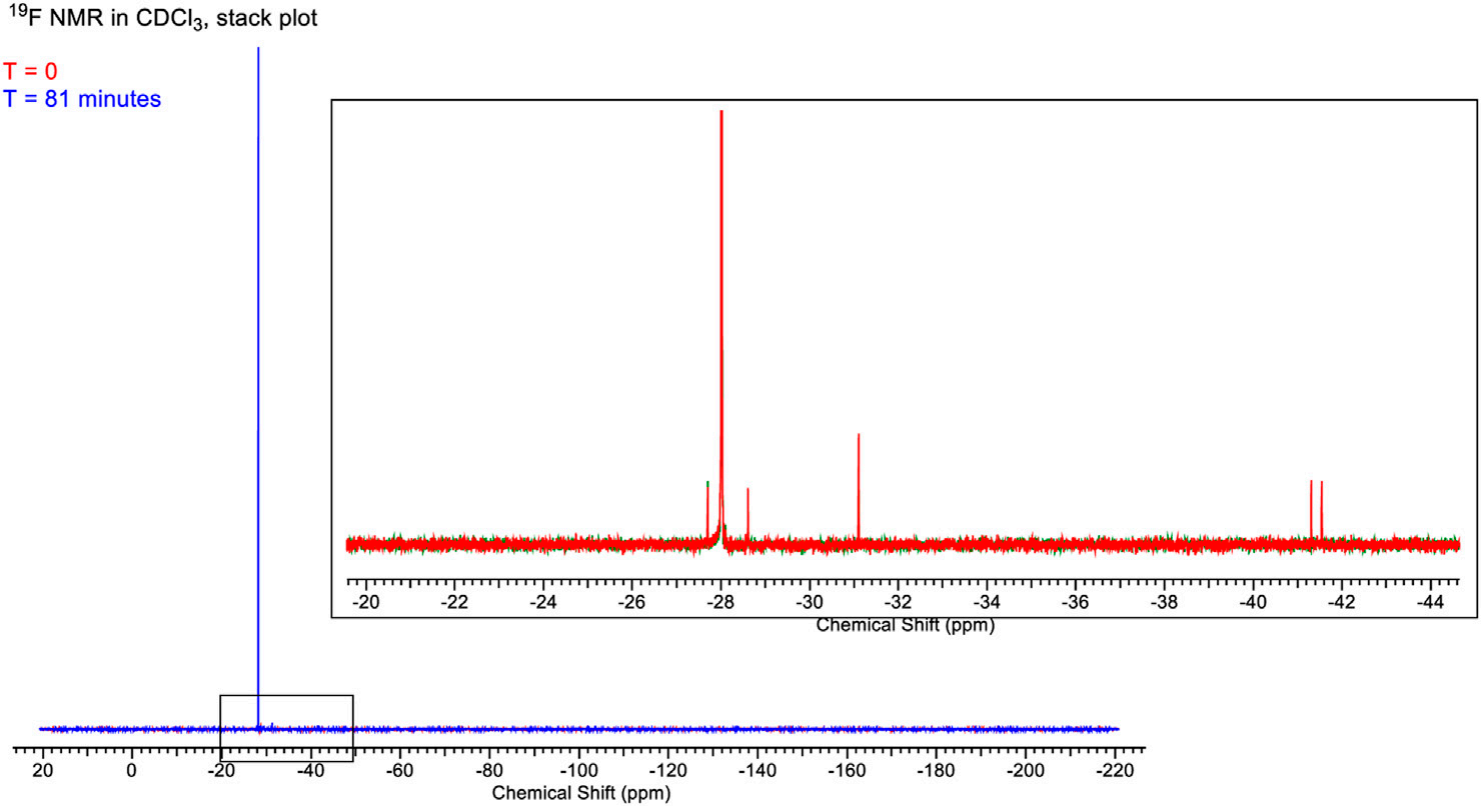
Full spectrum of the chemical shift of 4,5-dichloro-1,3-diethyl-imidazolylidene trifluoromethyl gold(I) in CDCl_3_ solution.

A set of ^1^H (Figure 13) and ^19^F (Figure 14) spectra were queued such that each element was monitored over 80 min. ^1^H NMR spectra are referenced to residual protio-solvent. The ^1^H spectra show no significant change over 81 min (time in the instrument, ∼5 min between sample creation and injection into the instrument). There was a slight drift in linewidths, and the resonance at δ1.5 ppm caused by water contamination broadens and shifts from 1.565 to 1.569 ppm, i.e., negligibly consistent with changes in H_2_O and HCl concentration. The image below shows the stacked spectra and a blown-up portion arising from the CH_3_ groups.

The ^19^F spectra also show minimal change over the time period. The stackplot shows only the first and last spectra recorded for clarity. The two spectra are essentially identical to save for the disappearance of two minor peaks at δ-41.5 ppm and δ-41.8 ppm. In the initial spectrum, these account for ∼2% of the total integrable intensity.

### Gibbs free energy of reaction

The Gibbs free energy (Salike and Bhatt, 2020; Lee et al., 2015; Martins and Cabral, 2019) of formation and reaction has been used multiple times to analyze the suitability of different complexes and organic or protein material for drug resistance (Tahir et al., 2020), equilibrium calculations of emulsion systems (Hosseini and Mohammadi, 2020), enthalpy of DNA formation (Lomzov et al., 2015), calculations of different mineral formation (Olivotos and Economou-Eliopoulos, 2016), and nanolithography (the present study). The Gibbs free energy of a compound is explained in terms of enthalpy (H), temperature (T), and entropy (S) by the relation: ΔG = ΔH–TΔS. The values of the enthalpy and entropy are taken from simulation data for each of the products and reactants. For a reaction, the Gibbs free energy is obtained (Ochterski, 2000) from relation (4) ΔG = G (products)–G (reactants), while for molecular complexes, relation (5) ΔG = G (species)–∑G (elements) suffices. The Gaussian software works in calculating the corrections to the enthalpy and entropy of formation or reaction based on total energy and contributions from vibrational, rotation, translational, or electronic motion; according to this, the enthalpy correction is explained by the relation (6): H_Corr_ = E_tot_ + k_B_T (Ye et al., 2018), where E_tot_ is the total energy. In a similar manner, the entropy is defined by S_tot_ = S_t_ + S_r_ + S_v_ + S_e_ (7) (Ye et al., 2018), where S_t_, S_r_, S_v_, and S_e_ are translational, rotational, vibrational, and electronic contributions, respectively.

Materials in powder form are usually analyzed for the moisture content mostly in the food industry (Lüttge, 2006; Cano-Higuita et al., 2015; López-Vidaña et al., 2021) and to obtain the dissolution rates of complexes (Zhang et al., 2019) through the calculation of the Gibbs free energy, but the use of Gibbs free energy still remains the means to determine the stability of a compound using simulation obtained values of entropies and enthalpies. In order to analyze the suitability of the Cl_2_ImEtCF_3_Au precursor, the Gibbs free energy from DFT calculations has been used. More industrial oriented applications to pipeline transport industry (water, gas, oil, and steam) (Jäger and Span, 2012) are by the analysis of Gibbs free energy of solid-state CO_2_ in the transport of CO_2_ in carbon capture and storage (CCS). The Gibbs free energy is the entity that defines the probability of a reaction to take place, the volatility and stability of the compound. The values of ε_0_, ε_ZPE_, H_Corr_, and G_Corr_ are calculated from the thermochemistry of Cl_2_ImEtCF_3_Au at the DFT level using B3LYP with a Def2-TZVPP basis set, where ε_0_ is the electronic energy, ε_ZPE_ is the zero-point energy, H_Corr_ is the enthalpy correction, and G_Corr_ is the Gibbs free energy correction. The sum of the electronic and enthalpy energy, the sum of the electronic and Gibbs free energy, and the sum of the electronic and zero-point energy are used as ε_0_ + H_Corr_, ε_0_ + G_Corr_, and ε_0_ + ε_ZPE_. The calculated thermochemistry values are presented in Table 5 with the values obtained from the Gaussian calculations. The calculations have been performed at a temperature of 298.15 K and a pressure of 1 × 10^-4^ Pa. The reaction with the result of an anion and a neutral fragment formation follows the pathway Cl_2_ImEtCF_3_Au^*^ → Cl_2_ImEtAu^−^ (*m/z* 389) + CF_3_ (*m/z* 69) for which the reaction energy and enthalpy are calculated to obtain the bond dissociation energy (BDE) and bond dissociation free energy (BDFE) of the reactants into products of reaction. Calculations of Gibbs free energy at the atomistic level with great results in modeling of crystal defects are reported by Cheng and Ceriotti (2018). Though at defect sites, the model predicts energies 300% higher than evaluated, and at non-defect sites, it predicts energies 10% higher than reported for the evaluated model. The higher defect estimated value of the Gibbs free energy is presented as a result of the anharmonicity at the defect sites with the transition at higher temperatures (>298 K). BDE (Fukaya et al., 2001; Luo, 2007; Woldu and Mai, 2012) and BDFE (Parr and Pearson, 1983; Moroz, 2011) are calculated for the chemical reaction, taking into account the ε_0_ correction to electronic energy and the enthalpy and Gibbs free energy corrections H_Corr_ and G_Corr_ at 298.15K, obtaining the change in enthalpy (4) and Gibbs free energy (3) with the reaction.

$$\Delta_{r}G=\sum {\left(\varepsilon_{0}+G_{Corr}\right)}_{products}-\sum {\left(\varepsilon_{0}+G_{Corr}\right)}_{reactants},$$ (3)

$$\Delta_{r}H=\sum {\left(\varepsilon_{0}+H_{Corr}\right)}_{products}-\sum {\left(\varepsilon_{0}+H_{Corr}\right)}_{reactants}.$$ (4)

**TABLE 5:** Free Gibbs energy correction, enthalpy correction, and zero-point corrections of Cl_2_ImEtCF_3_Au^−^, Cl_2_ImEtAu^−^, and CF_3_, respectively, which are products of formation and products of reaction.

	Cl_2_ImEtCF_3_Au^−^	Cl_2_ImEtAu^-^	CF_3_
**ε**_**0**_	−1776.5091596	−1439.2199272	−337.5510254
**ε**_**ZPE**_	0.176871	0.167681	0.012158
**E**_**tot**_	0.195293	0.183187	0.015612
**H**_**Corr**_	0.196237	0.184131	0.016556
**G**_**Corr**_	0.125203	0.120828	−0.014568
**ε**_**0**_ **+ ε**_**ZPE**_	−1776.332289	−1439.052246	−337.538867
**ε**_**0**_ **+ E**_**tot**_	−1776.313867	−1439.036740	−337.535414
**ε**_**0**_ **+ H**_**Corr**_	−1776.312922	−1439.035796	−337.534470
**ε**_**0**_ **+ G**_**Corr**_	−1776.383957	−1439.099100	−337.565594
**Reaction and formation products**	**Δ**_**r**_ **H (kcal/mol)**	**Δ**_**r**_ **G (kcal/mol)**	**S (kcal/mol)**
	−167.07443	−164.282	49.235

BDEs and BDFEs can be obtained for the formation reaction of the products of reaction, the anion and the neutral fragment Δ_f_H (5) and Δ_f_G (6):

$$\Delta_{f}H=\sum \Delta_{f}H_{products}\left(298K\right)-\sum \Delta_{f}H_{reactants}\left(298K\right),$$ (5)

$$\Delta_{f}G=\Delta_{f}H\left(298K\right)-T\;*\left(S{\left(298K\right)}_{parent}-\sum S{\left(298K\right)}_{fragments}\right).$$ (6)

The results of the calculations are presented in Table 5 with a reaction BDE of −0.2618 (Hartree: 164.282 kcal/mol).

The accuracy of the B3LYP/Def2-TZVPP basis set is related to spin-orbit coupling effects (Armbruster et al., 2006), where large errors are obtained in total atomic energies and atomization energies for heavy atoms (as is the case of the Au). The effective core potential (ECP) addition to the basis sets would reduce the effects induced in the 5p and 6p elements and the relativistic effects. The use of two component approaches is the most common method discussed in detail by Xu and Truhlar (2011), where (2p2s) polarization functions are added to the triple-ζ basis sets to reduce the aforementioned presented errors. A difference of 23.868 kcal/mol is obtained in the BDE values, from the reaction energy and DFT calculation. Large errors of over 10–12 kcal/mol (Armbruster et al., 2006; Xu and Truhlar, 2011) are known to be produced by high basis sets, such as TZVP, LANL2DZ, and Def2-XYVP (where XY = TZ, QZ, and so on).

The metal–ligand bond between Au(I) and CF_3_ presents higher BDE for Cl_2_ImEtCF_3_Au (189.15 kcal/mol) than for Au(I)–CF_3_ in CF_3_AuCO of 151.4 kcal/mol, Au(I)–Cl in ClAuPMe_3_ of 77.9 kcal/mol or Au(I)–Me in MeAuPMe_3_ of 43.4 kcal/mol reported by Marashdeh et al. (2017). The value reported from our calculations of −164.282 kcal/mol is specific for an exergonic process releasing energy, though it is not characterized by a high cross-section value for the elimination of the Au–CF_3_ ligand. Lower Δ_f_G would mean that the molecule is unstable, making it hard to work with and difficult to transfer from the vial through the gas line inside the vacuum chamber. Values as low as +16.5 kcal/mol for ClAuPF_3_ have been reported by Marashdeh et al. (2017), rendering the ClAuPF_3_ compound as one of the compounds with low vaporization pressure. Not stable in air and at room temperature, Cl_2_ImEtCF_3_Au has similar behavior to AuCF_3_CO (Marashdeh et al., 2017; Martínez-Salvador et al., 2011) that darkens in the presence of heat and light, a sign of the oxidation process. Cl_2_ImEtCF_3_Au is not to be kept at temperatures higher than 5°C as it spontaneously breaks ligands and degrades, while the presence of air would intensify the process of degradation and oxidation.

## Conclusion

4,5-Dichloro-1,3-diethyl-imidazolylidene trifluoromethyl gold(I) was analyzed for its suitability as a FEBID precursor. As a newly designed compound specifically for the deposition of nanoscale structure, its vaporization pressure, stability in air, and volatility have been studied using proton NMR and Gibbs free energy of reaction.

A good volatility value was obtained for the compound and a high stability in air with very low modifications of the structure during exposure. Its fragmentation, resonances, and anions at low electron energies and DEA have been obtained using velocity map imaging studies with great success. The structure, packing, orientation of the planes, and grain size have been run making use of powder XRD diffractometer data, and the VESTA simulation software has offered reliable insights into the crystalline vs. amorphous structure of the compound.

## Data Availability

The data presented in the study are deposited in https://www.ccdc.cam.ac.uk/structures/. The 4,5-dichloro-1,3-diethyl-imidazolylidene trifluoromethyl gold(i) compound was registered in the Cambridge Structural Database with the CCDC number 2223878.
